# In vitro multi-enzymatic cascades using recombinant lysates of *E. coli*: an emerging biocatalysis platform

**DOI:** 10.1007/s12551-020-00618-3

**Published:** 2020-01-20

**Authors:** Apostolos Alissandratos

**Affiliations:** 1 Research School of Chemistry, The Australian National University, ACT, Canberra, 2601 Australia; 2 CSIRO Synthetic Biology Future Science Platform, The Australian National University, ACT, Canberra, 2601 Australia

**Keywords:** Multi-enzymatic cascades, Cell-free biocatalysis, Cell-free synthetic biology, Cell-free metabolic engineering, Lysate, Cell-free extract

## Abstract

In recent years, cell-free extracts (or lysates) have (re-)emerged as a third route to the traditional options of isolated or whole-cell biocatalysts. Advances in molecular biology and genetic engineering enable facile production of recombinant cell-free extracts, where endogenous enzymes are enriched with heterologous activities. These inexpensive preparations may be used to catalyse multistep enzymatic reactions without the constraints of cell toxicity and the cell membrane or the cost and complexity associated with production of isolated biocatalysts. Herein, we present an overview of the key advancements in cell-free synthetic biology that have led to the emergence of cell-free extracts as a promising biocatalysis platform.

## Overview

Evolution has bestowed enzymes, nature’s catalysts, with remarkable catalytic prowess which is unrivalled by man-made catalysts (Schoemaker et al. 2003). Enzymes are able to carry out complex chemical transformations with very high enantio-, regio- and chemo-selectivity in benign aqueous solutions and under ambient temperature and pressure (Nestl et al. 2014; Sheldon and Brady 2019; Sheldon and Brady 2018). It becomes evident that biocatalysis has the potential to offer attractive green alternatives to many unsustainable fossil fuel-reliant chemical processes that we have come to depend upon for fuel, food, materials and medicines (Bornscheuer et al. 2012; Bornscheuer 2018).

An additional advantage is that enzymes are highly compatible with one another, even when sourced from unrelated organisms (France et al. 2017; Hold et al. 2016). This facilitates employment of many enzymes in tandem for single-pot, multistep transformations of simple building blocks into complex chemical structures. Indeed, enzymes from bacteria, archaea, insects and mammals may be readily mixed to produce target chemicals through artificial biosynthetic pathways. The great potential of this approach has been demonstrated through several impressive examples of in vitro multi-enzymatic cascades (Korman et al. 2017; Rollin et al. 2015; Wang et al. 2017). Notably, Williamson and co-workers (Schultheisz et al. 2008; Schultheisz et al. 2010) reconstituted the entire de novo purine and pyrimidine biosyntheses to produce labelled nucleoside triphosphates (NTPs) from simple feedstocks (e.g. bicarbonate and canonical amino acids). This is in stark contrast to chemical methodologies that rely on toxic reagents and multiple protection-deprotection cycles and elaborate purifications of intermediates and products (Burgess and Cook 2000).

Despite these advantages, examples of successful in vitro employment of enzymes in industrial processes have been fairly limited. Though reasons for this are complex and case-specific, an important common contributing factor is the expense of biocatalyst separation which may account for the majority of total production cost (Tufvesson et al. 2011). In multi-enzymatic processes, this expense increases proportionally with the number of enzymes that must be produced and isolated separately. Further to this, enzymes have evolved to rely on various cofactors that are necessary for their catalytic activity (Zhao and van der Donk 2003). An important subset of cofactors are small organic molecules such as ATP and NAD(P)H that are also referred to as co-substrates because they are typically spent during anabolic reactions and regenerated through catabolic processes (Swartz 2012). However, when a cofactor-dependent enzyme is removed from this metabolic context, it must then be supplied in stoichiometric amounts. The high cost and low stability of such compounds prohibit economical employment as reagents, thus limiting ATP-/NAD(P)H-dependent biocatalysis.

On the other hand, whole-cell bioprocesses with native or engineered cells involve multistep enzymatic transformations without the requirement for protein purification or supply of cofactors (France et al. 2017). Though the whole-cell approach is effective for many fermentative biotransformations, it is not readily applicable to just any multi-enzymatic cascade. Within the cell, the reaction of interest is confounded with the side activities of cell propagation and growth and other important metabolic functions (Billerbeck et al. 2013). Control over multistep metabolic routes is limited, often resulting in bottlenecks and losses due to siphoning of intermediates for the production of undesirable side products. Most importantly, toxic substrates, intermediates and/or products cannot be employed in living cells, and even natural metabolites are often only tolerated within relatively narrow concentration ranges.

**Fig. 1:**
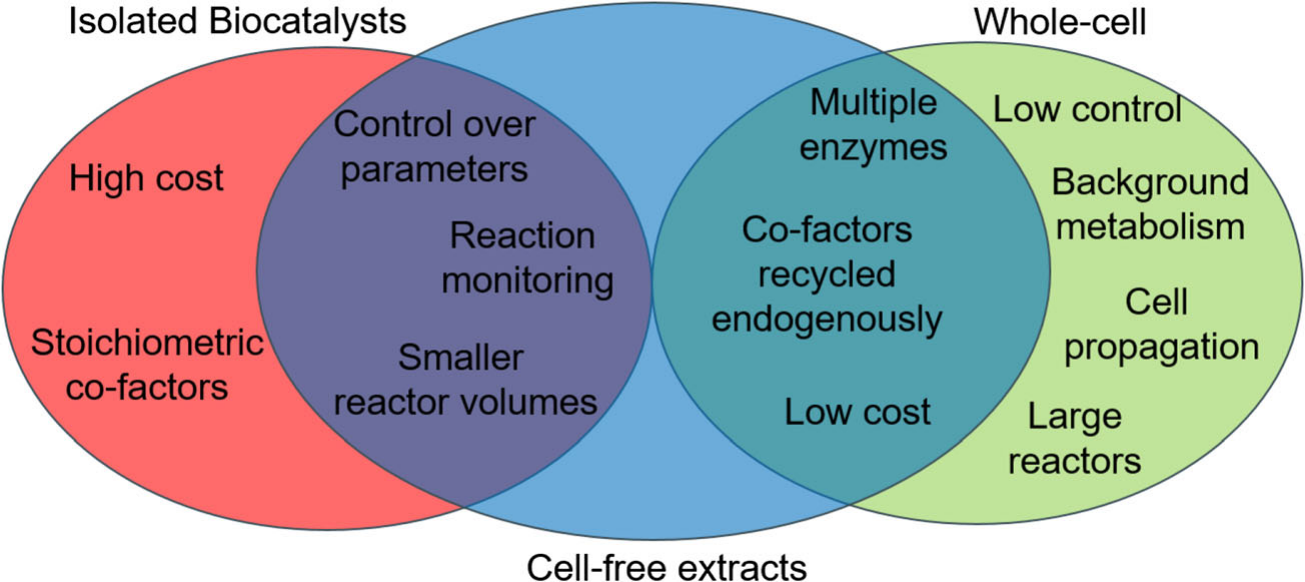
Cell-free extracts offer a third route for the application of biocatalysts combining many of the benefits of isolated biocatalysts and whole-cell systems

A third route to biocatalysis has emerged in recent years (Fig. 1), gaining considerable momentum with the advent of synthetic biology and the ever increasing availability of methodologies for straightforward genetic engineering of well-understood biotechnological organisms, such as *E. coli*. This involves the use of cell-free extracts (or lysates; though cell-free extract might imply some additional processing of the clarified lysate, this is typically minimal and therefore the terms are used interchangeably) prepared from engineered recombinant cells as an inexpensive source of all requisite enzymatic activities (Swartz 2018). Through this approach, endogenous enzymes that are naturally abundant in a lysate may be used to support recombinantly overexpressed enzymes in carrying out multistep biochemical reactions. Importantly, as the recombinant enzymes are produced in the same cells that provide native endogenous activities, all necessary enzymes may be supplied from a single preparation, produced from a bacterial culture with minimal processing (lysis-clarification). In addition, endogenous enzymes or entire central metabolic pathways may be recruited for cofactor recycling, thus overcoming another important limitation of in vitro biocatalysis with no requirement for additional exogenous enzymes. On the other hand, the absence of a cell membrane removes transport limitations and allows increased control over reaction parameters. Substrate and catalyst loadings may be straightforwardly optimised while toxicity is no longer a limiting factor. This approach is also referred to as cell-free metabolic engineering (Swartz 2018) as, in addition to the introduction of heterologous activities, the metabolic profile of the cell used to produce the extract may be engineered further, e.g. through gene deletions or engineering of homologous host genes. The following is not intended to be an exhaustive review of a highly interdisciplinary area with often hard-to-define boundaries, but rather an overview of advancements that have led to the emergence of cell-free extracts as a viable platform for the catalysis of multistep cascades.

## Background

Early enzymologists lacked straightforward methods for the purification of their target proteins. Crude cell extracts were therefore invaluable tools, routinely employed for the deciphering of enzyme mechanism and properties. In fact, Eduard Buchner’s description of a cell-free alcoholic fermentation using yeast cell extract (Buchner 1897) constitutes one of the earliest and most important discoveries in the field of biochemistry. Modern molecular biology and more specifically the advent of recombinant DNA technologies have provided straightforward access to almost any desired protein, tagged with a small peptide (e.g. hexahistidine-tag) for separation with a single affinity column (Kimple et al. 2013). Ease-of-access to pure recombinant proteins at lab-scale meant that cell-free extracts became limited to niche applications, predominantly cell-free protein synthesis. However, it was quickly realised that endogenous lysate enzymes could be recruited to support cell-free protein synthesis, e.g. through provision of “chemical energy” (Calhoun and Swartz 2005b), synthesis of expensive ingredients (e.g. NTPs) (Alissandratos et al. 2016; Calhoun and Swartz 2005a) or in situ deprotection of otherwise labile unnatural amino acids (Arthur et al. 2013). Increased research activity into cost-efficient cell-free protein synthesis in recent years has ultimately fuelled the re-emergence of cell-free extracts as a viable biocatalysis platform, now further bolstered with straightforward genetic engineering methodologies and technological advancements in high-throughput automation and analysis.

## The importance of cofactor recycling

**Fig. 2:**
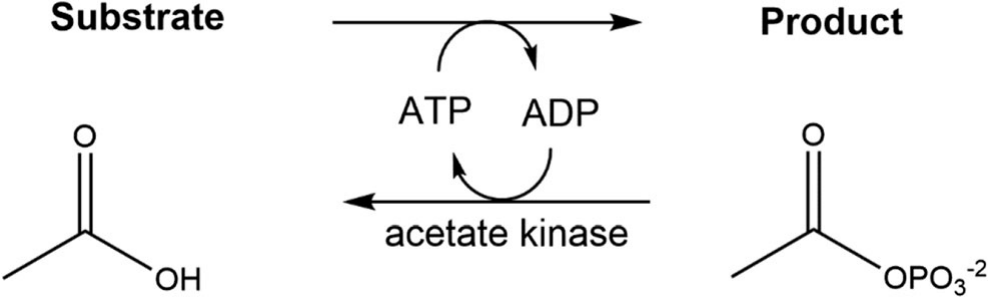
Cofactor recycling through an auxiliary enzymatic reaction (acetate kinase) with an inexpensive substrate (acetyl phosphate)

As discussed, in vitro application of enzymes is often accompanied by a requirement for the supply of organic cofactors, such as ATP and NAD(P)H. In in vitro reactions, these expensive compounds act as co-substrates and are required in prohibitive stoichiometric amounts. Biochemists have overcome this obstacle by coupling the reaction of interest with an auxiliary enzymatic reaction which regenerates the spent cofactor using an inexpensive substrate (Fig. 2) (Zhao and van der Donk 2003). This ensures a constant supply of the necessary cofactor when employed in catalytic amounts, thus also avoiding any inhibitory effects of high cofactor concentrations (e.g. substrate inhibition). Whitesides pioneered this area in the 1980s and 1990s by reporting an array of useful ATP-recycling reactions and methods for straightforward synthesis of the necessary substrates, with acetate kinase/acetyl phosphate and pyruvate kinase/phosphoenol pyruvate being the most popular (Chenault et al. 1988; Crans et al. 1987). Though a wide range of auxiliary enzyme/substrate pairs have been proposed over the years for almost all cofactors, each with its own advantages and disadvantages, all share the requirement for at least one additional isolated enzyme (Chenault et al. 1988; Zhao and van der Donk 2003).

Seminal work in cell-free protein synthesis applications identified the possibility of utilising endogenous enzymes, already present as inert contaminants in the cell-free extract, to catalyse cofactor recycling. Spirin and co-workers reported that the requirement of ribosomal protein synthesis for ATP and GTP could be met through endogenous recycling with the same inexpensive substrates proposed by Whitesides (Ryabova et al. 1995). Swartz and co-workers reported the use of glycolytic intermediates or even glucose itself, to support these requirements (Calhoun and Swartz 2005a; Calhoun and Swartz 2005b; Kim and Swartz 2001). This culminated in the production of the Cytomim system, a cell-free extract tailored to closely mimic the cytoplasmic environment (Jewett et al. 2008). This was described in a key publication, which summarised the remarkable ability of endogenous cell-free extract metabolism to balance necessary cofactors, provide costly reagents (NTPs from monophosphates) and how physiochemical parameters (e.g. pH, ionic strength) may be perturbed to prolong protein synthesis and optimise productivity and cost-efficiency.

## Cascades incorporating central metabolism

**Fig. 3:**
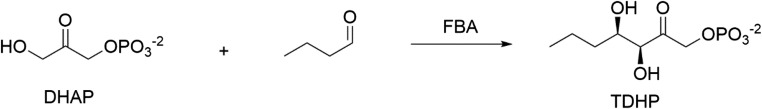
Reaction employed by Bujara et al. (2010) for the conversion of butanal- and glucose-derived dihydroxyacetone phosphate (DHAP) into 5,6,7-trideoxy-D-threoheptulose-1-phosphate (TDHP) through the action of fructose-bisphosphate aldolase (FBA)

The realisation by Swartz and co-workers that endogenous central metabolism and glycolysis in particular were highly active in the *E. coli* cell-free extract also constituted examples of cell-free extract-catalysed multistep transformations. Yet these reactions employed exclusively endogenous enzymes and were carried out in the context of cell-free protein synthesis to support and extend ribosomal productivity. Panke and co-workers utilised the same endogenous glycolytic cascade but specifically for the biochemical synthesis of dihydroxyacetone phosphate and its further conversion (Fig. 3) into vicinal diols by aldolases (Bujara et al. 2010). Through a series of papers, the Panke group demonstrated the great potential of their approach as well as the advantages conferred by the coupling of cell-free biocatalysis with recent technological advancements (Billerbeck et al. 2013; Bujara et al. 2010; Bujara et al. 2011). Importantly, the absence of the cell membrane conferred increased control over reaction conditions and allowed on-line mass spectrometric monitoring of metabolites for the avoidance of bottlenecks and the overall optimisation of productivity.

In addition to glycolysis, Swartz and co-workers showed that the pentose phosphate pathway was also active in native *E. coli* extract and could be employed to regenerate NADPH from glucose (Lu et al. 2015). This system was coupled to a synthetic in vitro cascade using isolated ferredoxin NADP^+^ reductase, ferredoxin and a ferredoxin-dependent [FeFe] hydrogenase for the conversion of NADPH and H^+^ to hydrogen. Coupling the isolated biocatalyst reaction to the extract-catalysed reaction allowed single-pot production of hydrogen from glucose*.* Scrutton and co-workers described the application of cell-free extracts containing overexpressed recombinant NADPH-dependent enzymes, for the conversion of pulgenone to menthol (Toogood et al. 2015). In this work, it was also noted that use of endogenous *E. coli* NADPH recycling with glucose was able to support menthol synthesis; however an exogenous NADPH recycling system improved yields significantly.

**Fig. 4:**
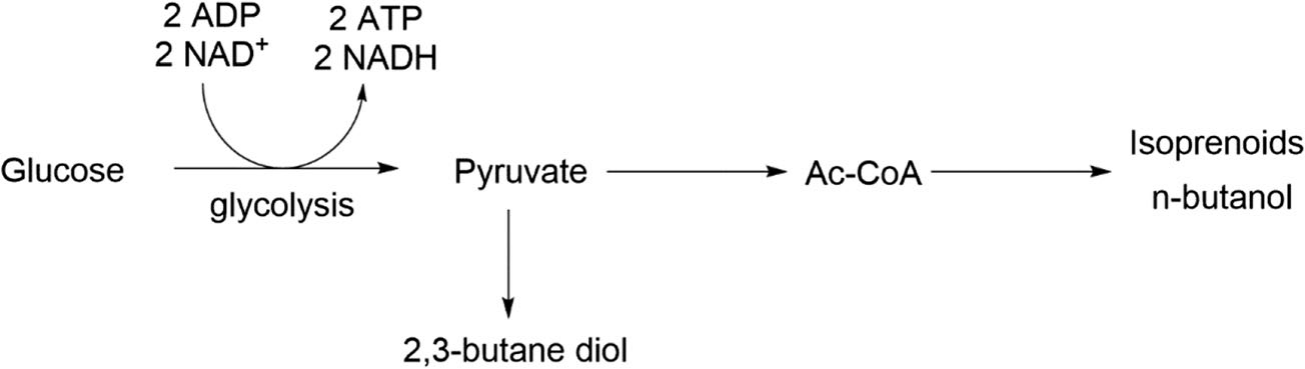
Cell-free extract catalysed production of platform chemicals and isoprenoids through coupling of recombinant activities to endogenous glycolysis which also supplies cofactors

At around the same time, Kay and Jewett published their seminal work on lysate-catalysed production of 2,3-butanediol from glucose (Kay and Jewett 2015). This system relies on endogenous glycolytic activity for the production of pyruvate which is further transformed through a three-step recombinant enzyme cascade (Fig. 3). Importantly, the recombinant enzymes were co-expressed in the same *E. coli* cells used to produce the cell-free extract. In this way, all endogenous and recombinant enzymes necessary for the full 13-step transformation could be prepared from a single bacterial culture. High conversions of glucose (74%) were achieved with ~900 turnovers of the NADH cofactor necessary for the final reduction of acetoin to 2,3-butanediol. The same group also employed this approach for the production of mevalonate, an important intermediate in the synthesis of isoprenoids (Fig. 4) (Dudley et al. 2016). Transformation of glucose to pyruvate and then acetyl-CoA was supported by endogenous enzymes, while production of mevalonate from acetyl-CoA was catalysed by recombinant enzymes. In this and other work (Casini et al. 2018; Karim and Jewett 2016), the Jewett group have also demonstrated in situ production of the necessary recombinant enzymes through cell-free protein synthesis, exploiting the presence of native ribosomal machinery. Furthermore, through the combinatorial mixing of lysates, each expressing a single recombinant enzyme, prototyping of multistep heterologous metabolic pathways was possible. This was applied for the conversion of glucose-derived acetyl-CoA to n-butanol using recombinant *Clostridium acetobutylicum* enzymes (Karim and Jewett 2016), while in a separate example, mevalonate biosynthesis was extended for the production of limonene to complete the isoprenoid biosynthesis pathway (Dudley et al. 2019).

## Beyond glycolysis

**Fig. 5:**
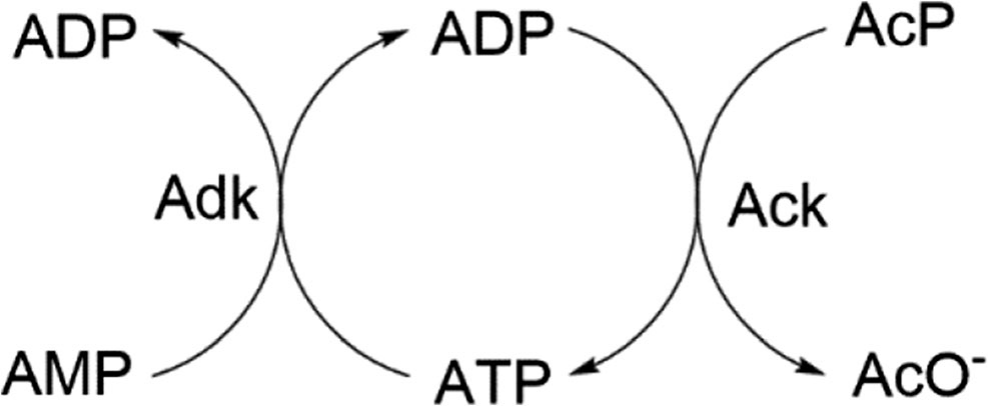
ATP recycling system based on the activity of endogenous lysate adenylate kinase (Adk) and acetate kinase (Ack) with acetyl phosphate (AcP)

In the above examples, multi-enzymatic transformations benefit from the abundance of endogenous lysate glycolytic enzymes that catalyse the transformation of glucose as well as balance cofactor requirements. Nonetheless, cell-free extracts of *E. coli* have also been employed to carry out cofactor-dependent multi-enzymatic cascades that do not intersect with such key metabolic processes. Advantageously, by circumventing important metabolic intersections, the loss of intermediates may be minimised, allowing quantitative yields with minimal optimisation. We have exploited the abundance of endogenous acetate kinase and adenylate kinase in *E. coli* lysates for rapid regeneration of ATP from AMP with acetyl phosphate (Fig. 5) (Alissandratos et al. 2016; Hennessy et al. 2018). The acetyl phosphate is prepared through simple procedures from cheap and readily available inorganic phosphate and acetic anhydride in aqueous solution which may be employed directly in enzymatic reactions (Alissandratos et al. 2016; Crans and Whitesides 1983). This system has been very useful for the synthesis of nucleotides from cheaper and more stable materials. More specifically natural and unnatural NTPs were prepared from monophosphates (Alissandratos et al. 2016), non-phosphorylated (deoxy)nucleosides (Alissandratos et al. 2016; Loan et al. 2019a) or even simple non-phosphorylated sugars and nucleobases (Loan et al. 2019b). In all cases, a recombinant lysate prepared from a single bacterial culture was the only required biocatalyst.

**Fig. 6:**
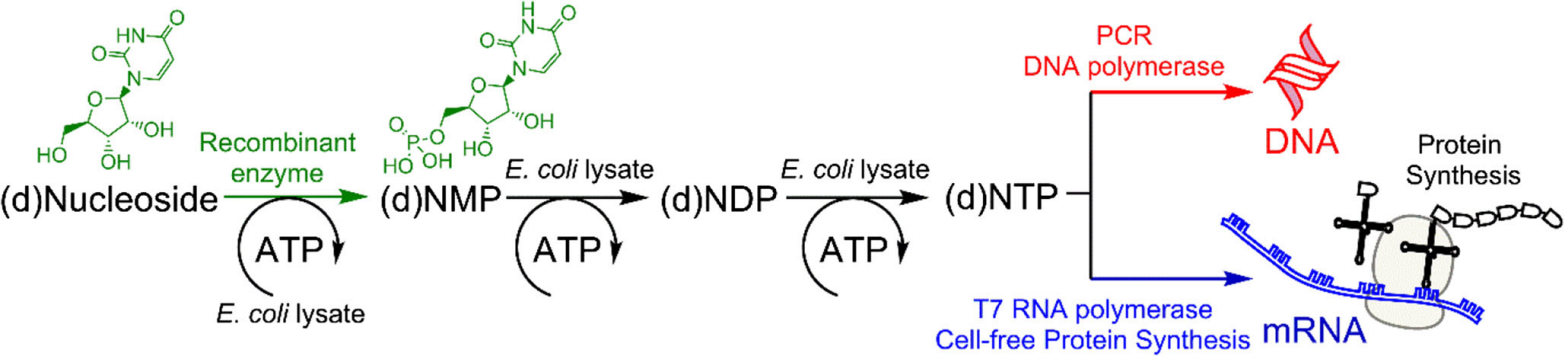
NMP phosphorylation by endogenous *E. coli* lysate kinases is coupled to a recombinant kinase for the production of NTPs. Alternatively, (d)NTPs produced from (d)NMPs are employed directly by polymerases for the synthesis of nucleic acids. Reprinted with permission from *Alissandratos* et al.*, ACS Chem. Biol. 2016, 11, 12, 3289–3293*. Copyright 2016 American Chemical Society

This was initially employed for the synthesis of (d)NTP mixtures for direct use in biotechnologically important polymerase-catalysed syntheses of mRNA (cell-free protein synthesis) or DNA (PCR) (Fig. 6) (Alissandratos et al. 2016). Endogenous synthesis of NTPs from NMPs was also coupled to recombinant uridine/cytidine kinase activity for the production of UTP, CTP and fluoro-UTP from the respective nucleosides. More recently, a single low-cost recombinant lysate was developed, containing all enzymes necessary to carry out DNA amplification with in situ conversion of unphosphorylated deoxynucleosides to dNTPs (Loan et al. 2019a). Through this approach, commercial cold-stored reagents (dNTPs, enzymes) that hinder de-centralised employment of important nucleic acid amplification (NAA) applications (e.g. diagnostics) are replaced by an inexpensive lysate-based preparation. This method holds great promise for simplified application of NAA within low-tech settings.

Recently, the nucleotide synthesis cascade was extended further for the production of uridine triphosphate (UTP) in g L^−1^ h^−1^ yield, from simple and inexpensive feedstocks, namely, sugar and nucleobase (Loan et al. 2019b). For this, a single recombinant lysate catalysed the quantitative transformation of equimolar starting concentrations of ribose and orotic acid into UTP, through a partial de novo pyrimidine biosynthesis cascade coupled to ribose phosphorylation. Importantly, for this system, the addition of acetyl phosphate to the reaction mix led to spontaneous generation of catalytic amounts of ATP from endogenous lysate materials. Therefore, no exogenous supply of cofactor was necessary to drive the four ATP-dependent steps of the pathway.

**Fig. 7:**
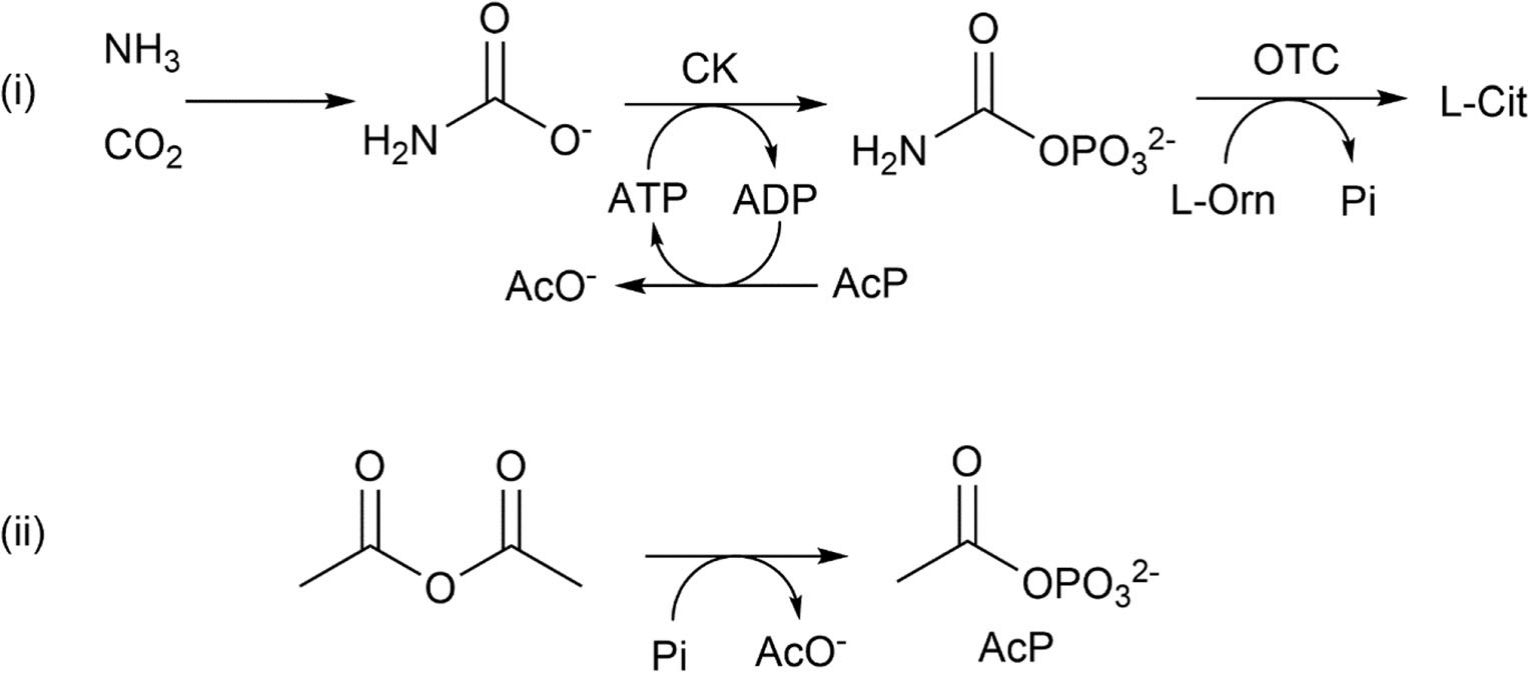
(**a**) Citrulline (L-Cit) synthesis by a single recombinant cell-free extract with recombinant carbamate kinase (CK) and ornithine transcarbamoylase (OTC) for the carbamylation of ornithine (L-Orn) to nitrogen-dense citrulline and (**b**) chemical synthesis of acetyl phosphate (AcP) from inorganic phosphate (Pi) and acetic anhydride.

The utility of this approach is not limited to the synthesis of nucleotides and nucleic acids. Recently, a cell-free extract was developed for catalysis of an in vitro synthetic cascade that transforms pervasive environmental pollutants (ammonia and carbon dioxide) into nitrogen-rich citrulline (Alissandratos et al. 2019). The reported cascade (Fig. 7) includes an initial chemical step for the formation of carbamate (spontaneous in aqueous mixtures of ammonia and carbon dioxide) which is then activated through phosphorylation by an ATP-dependent archaeal carbamate kinase for subsequent carbamylation of ornithine by an archeal transcarbamoylase. The cascade is driven by the acetyl-phosphate-based endogenous recycling of ATP to generate g L^−1^ h^−1^ citrulline. As before, the acetyl phosphate is produced from acetic anhydride and inorganic phosphate in a separate reaction (Fig. 7). Importantly, in this case, in vitro biocatalysis is necessary to enable the initial non-enzymatic step. Ammonia conversions of > 90% in 90 min were achieved through simple optimisation of catalyst loading, for ammonia concentrations that were representative of levels found in municipal wastewater. Remarkably the same biocatalyst showed no decrease in activity for 1000-fold more concentrated ammonia solutions, such as those found in heavily polluted industrial wastewaters. Ammonia solutions used to scrub carbon dioxide from simulated industrial flues were also suitable feedstocks for the cell-free extract. Lastly, citrulline was found to be a promising nitrogen plant fertiliser, offering a renewable alternative to widely utilised fertilisers (e.g. urea) that are produced through unsustainable processes.

## Future outlook

The ever-growing interest in cell-free synthetic biology and the accompanying development of new tools and methodologies holds great promise for future application of cell-free extract biocatalysts (Bundy et al. 2018; Dudley et al. 2015; Hodgman and Jewett 2012; Swartz 2012; Wilding et al. 2018). The development of new cell-free platforms from organisms such as the fast-growing bacterium *Vibrio natriegens* (Des Soye et al. 2018) will expand the scope of biocatalytic transformations that may be carried out. Further advances in genetic engineering and the design of bespoke genetic elements will allow tighter regulation of the rate and yield of protein expression. This will lead to increased control over the enzymatic profile of the cell-free extract and avoidance of side-reactions and bottlenecks. The rapid expansion of cell-free extracts into new areas (biomanufacturing, diagnostics, pharmaceuticals) (Bundy et al. 2018; Loan et al. 2019a; Pardee et al. 2016a; Pardee et al. 2016b; Silverman et al. 2019; Slomovic et al. 2015) is set to offer exciting opportunities for the interfacing of traditional biocatalysis with other cell-free synthetic biology applications. In this direction, the Jewett group have already demonstrated cell-free production of important glycoproteins and non-ribosomal peptides through the combination of cell-free protein synthesis and biocatalysis (Goering et al. 2017; Jaroentomeechai et al. 2018; Kightlinger et al. 2019).

The application of cell-free extracts in industrial biocatalysis will require readily scalable straightforward production methodologies and stable formulations. Cell-free extracts are already generated as intermediates in the production of isolated commercial biocatalysts; therefore the necessary bioprocesses are already available at scale (Tufvesson et al. 2011). The cheaper cell-free extracts may be less stable than isolated enzymes due to the presence of background proteolytic activities and other contaminants, though this may be addressed through appropriate use of inhibitors and engineered strains. In addition, straightforward lyophilisation methods allow the production of room temperature stable cell-free extract formulations (Smith et al. 2014; Pardee 2018). Bundy and co-workers have described detailed methodologies for the preparation of cell-free extracts that retain activity over months (Smith et al. 2014; Wilding et al. 2019), thus holding great promise for commercial applications. Cell-free extracts might be considered more suitable for homogeneous catalysis through batch biocatalytic processes, mainly due to the technical challenges associated with immobilising the mixtures of cell-free extract components. Nonetheless, continuous cell-free protein syntheses have been carried out with commercially available ultrafiltration devices and membranes (Spirin et al. 1988). This approach may also form the basis for future efforts in the development of continuous biocatalytic processes with cell-free extracts.

All in all, the future appears bright for this emerging cost-efficient biocatalysis platform that has already been applied successfully for the synthesis of an array of important platform and fine chemicals. Nonetheless, widespread application of such cell-free systems will also require a better understanding of the physiochemical interactions involved. As recently illustrated, changes in reaction parameters may strongly impact the performance of cell-free extract-catalysed transformations (Karim and Jewett 2016; Karim et al. 2019). It is our hope that this review will highlight the great potential of cell-free extracts in biosynthesis and inspire biophysicists to further explore the biological, chemical and physical complexity of these systems.
